# Sub-Tenon’s Block in Patients with Previous Encircling Band Surgery—A Feasibility Study

**DOI:** 10.3390/jcm13247735

**Published:** 2024-12-18

**Authors:** Johannes Harte, Gesar Ugen, Joana Berger-Estilita, Andreas Ebneter, Friedrich Lersch

**Affiliations:** 1 Department of Anesthesiology and Pain Medicine, Bern University Hospital, Inselspital, 3010 Bern, Switzerland; 2 Institute for Medical Education, University of Bern, 3012 Bern, Switzerland; 3 CINTESIS@RISE, Centre for Health Technology and Services Research, Faculty of Medicine, University of Porto, 4200-450 Porto, Portugal; 4 Department of Ophthalmology, Cantonal Hospital St. Gallen, 9007 St. Gallen, Switzerland

**Keywords:** sub-Tenon, buckling surgery, encircling band, regional anesthesia

## Abstract

**Introduction:** During the COVID-19 pandemic, reducing aerosol-generating procedures became fundamental, particularly in ophthalmic surgeries traditionally performed under general anesthesia (GA). Regional anesthesia, such as sub-Tenon’s block (STB), is widely used in vitreoretinal surgeries, offering a safer alternative by avoiding airway manipulation. However, the altered orbital anatomy in patients with previous scleral explant surgery creates unique challenges to STB application. This study aims to evaluate the effectiveness, safety, and feasibility of STB in patients after encircling band surgery. **Methods:** This retrospective analysis included 46 patients with a history of scleral explant surgery, undergoing vitreoretinal procedures at the Bern University Hospital. All procedures were conducted under STB with either analgosedation or GA for additional support when required. An ophthalmic surgeon or an experienced anesthesiologist performed the STBs. Data collected included block success rate, procedural difficulty, incidence of chemosis, and patient satisfaction. The Institutional Ethics Committee approved this study, and all participants provided informed consent. **Results:** STB was successfully administered in 93.5% of cases, with only three unsuccessful blocks. Block placement was rated as easy in 55% of cases, moderately difficult in 28%, and difficult in 17%. Chemosis was observed in 24% of patients, with severe cases in only 4%. Patient satisfaction scores were high, with most patients expressing satisfaction with the STB procedure. Conversion to GA was required in only one case due to alcohol withdrawal-related agitation. **Discussion:** The high success rate and minimal complications suggest that STB is a feasible and safe alternative to GA in patients with prior scleral buckling surgery. The altered orbital anatomy presents potential challenges, including scar tissue and compartmentalization, which may lead to patchy anesthesia. However, the use of STB avoids the risks associated with GA and may be especially beneficial for elderly or frail patients. Future studies should further investigate the hemodynamic implications of STB in these cases and the potential for ultrasound-guided techniques to improve accuracy and safety.

## 1. Introduction

The COVID-19 pandemic prompted healthcare providers to adopt protocols that limit aerosol-generating procedures, particularly in surgeries where proximity to the respiratory tract increases the risk of viral transmission [1]. In ophthalmic surgery, this need translated into an increased emphasis on regional anesthesia to avoid general anesthesia (GA) where possible [2,3]. Sub-Tenon’s block (STB) is a reliable and safe option for locoregional anesthesia in most vitreoretinal (VR) surgeries, providing excellent anesthesia and analgesia while eliminating the risks associated with airway manipulation in GA [4,5]. However, its use in patients with prior scleral explants in place is met with caution, as the altered orbital anatomy and presence of the encircling band can restrict the distribution of anesthetics and complicate block administration [6,7].

Traditionally, general anesthesia has been recommended in cases involving prior scleral buckling surgery due to concerns about complications from distorted ocular anatomy and the potential for ineffective blocks. The insertion of an encircling band often causes elongation or slight alteration in the structure of the eye, creating unique challenges for regional anesthesia, particularly when using sharp-needle techniques like retrobulbar or peribulbar blocks [8]. These blocks carry a heightened risk of globe perforation or other complications in elongated or scarred eyes [9]. STB, a blunt cannula technique that injects anesthetic into the sub-Tenon’s space, is less invasive and may mitigate some of these risks. However, due to the mechanical obstruction posed by the encircling band, the procedure can become technically challenging, and it is unclear whether successful anesthesia can consistently be achieved in such cases.

Prior to the pandemic, STB was selectively employed for VR surgeries in elderly or medically frail patients, who would face higher risks under GA. This approach was guided by clinical judgment, balancing the need for adequate anesthesia against the patient’s overall medical condition. During the pandemic, however, we began to extend this practice to a broader patient population with prior encircling scleral bands, aiming to minimize exposure to GA and reduce overall perioperative risk. In this feasibility study, we aimed to systematically evaluate the success rate, safety, and patient satisfaction associated with STB in patients who had previously undergone encircling band procedures. We hypothesized that STB could be a safe and effective alternative to GA, even in eyes with altered anatomy due to encircling bands. Our findings aim to inform future practice guidelines and contribute to expanding the application of regional anesthesia in complex ophthalmic cases, particularly in settings where GA poses additional risks.

## 2. Methods

### 2.1. Ethics

This study adhered the World Medical Association Declaration of Helsinki Ethical Principles for Medical Research Involving Human Subjects and complied with the Swiss Human Research Act. The Cantonal Ethics Committee of Bern (KEK Bern) Switzerland (Chairperson Prof. em. Dr. med. Christian Seiler) approved this study (BASEC-number: Req-2020-01360). Patients provided informed consent for both the sub-Tenon’s block procedure under analgosedation and the ophthalmic surgery itself. Additionally, patients consented to the anonymised data analysis and the subsequent publication of the study results.

### 2.2. Study Design

This study is a retrospective cohort analysis that examines outcomes of patients with prior encircling band surgery who received sub-Tenon’s block for vitreoretinal procedures. We included patients with a history of encircling scleral band placement who underwent various VR procedures, such as pars plana vitrectomy, oil removal, cryotherapy, and complex phacoemulsification, at the University Hospital of Bern, Switzerland.

Eligible Patients had a history of encircling scleral band placement. Participants were required to be scheduled for VR surgery, including procedures such as pars plana vitrectomy, oil removal, cryotherapy, and complex phacoemulsification, all of which necessitated a reliable anesthesia technique due to their complexity.

The ophthalmic surgeon and the anesthesiologist independently assessed the feasibility of performing STB. This evaluation considered factors such as the patient’s orbital anatomy, any scarring from previous surgeries, and the specific requirements of the planned procedure. Additionally, patients were selected if their general health condition made them suitable for STB with or without analgosedation. Priority was given to individuals who could benefit from avoiding general anesthesia due to underlying comorbidities or frailty, thereby minimizing perioperative risk.

All patients with severe eye infections, systemic contraindications to regional anesthesia, and with extensive conjunctival or Tenon’s capsule scarring were excluded due to the high likelihood of block failure or complications. Finally, we excluded all patients who were unable to provide informed consent.

The data for this study were collected retrospectively over two years, from March 2020 to April 2022, at the Department of Ophthalmology, University Hospital of Bern (Inselspital), Switzerland.

### 2.3. Outcome Measures

Primary Outcome: The success rate of STB, defined by adequate anesthesia during the procedure without the need for opioid administration in cases combined with GA.

Secondary Outcomes: Patient satisfaction rated on a scale, technical difficulty of block placement, incidence of adverse events, and overall effectiveness of the anesthesia.

#### 2.3.1. Intervention

Given their prior encircling band surgery, each patient received an STB tailored to their specific surgical and anatomical needs. Depending on availability and expertise, the intervention was administered by either an ophthalmic surgeon or an experienced anesthesiologist. The STBs were administered either by a vitreoretinal surgeon or an experienced anesthesiologist. The surgeon had five years of experience as a consultant in vitreoretinal surgery, following specialized ophthalmology training. The anesthesiologist had six years of experience leading the ophthalmology anesthesiology team, with extensive daily expertise in performing and teaching ophthalmic regional anesthesia. The process involved several standardised steps to ensure consistency in the procedure and evaluate technical challenges. First, patients were positioned for optimal access to the eye undergoing surgery. The surgeon or anesthesiologist identified the area for block placement based on the presence of the encircling scleral band. Careful attention was paid to avoid interference from the band and any scar tissue from previous surgery.

**Figure 1:**
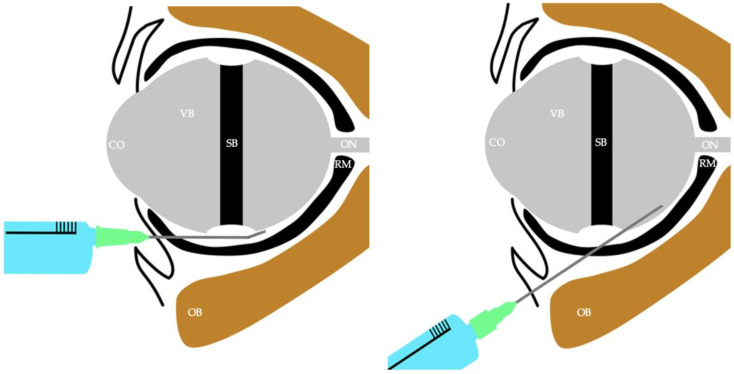
Comparing sub-Tenon block (STB) cannula trajectory vs. retrobulbar needle trajectory in a previously encircled, elongated eye; bulbar length is a serious risk for sharp-needle injury. The retrobulbar block, in contrast to the STB, penetrates the sclera. Legend: CO = cornea, VB = vitreous body, SB = scleral buckle, ON = optical nerve, RM = rectal muscle, OB = orbital bone.

After thorough disinfection, we opened the eye using a non-toothed forceps. Next, we lifted the conjunctiva with tweezers and made an incision in the conjunctiva and Tenon’s capsule using scissors. We then advanced a blunt, pre-shaped 22G needle into the sub-Tenon space dorsally behind the eye, always following the contour of the globe. The sub-Tenon’s space was typically accessed in the inferonasal quadrant to allow better distribution of the anesthetic while minimizing the risk of complications (watch the instructive video in the Supplement or consult Figure 1. For patients with scar tissue from previous surgeries, the clinician took additional time and care in positioning the cannula to avoid resistance or damage. Five mL of local anesthetic (a mixture of ropivacaine 1%, hyaluronidase 5 IU/mL, and clonidine 5 µg/mL) was injected into the sub-Tenon’s space through the cannula, aiming at distribution behind the scleral buckle. This combination was selected based on its clinical benefits: ropivacaine 1% provides long-lasting anesthesia and analgesia, hyaluronidase 5 IU/mL facilitates the spread of the anesthetic within tissue planes, and clonidine 5 µg/mL prolongs the duration of analgesia through its α2-adrenergic agonist properties. During administration, the clinician monitored for any indications of restricted anesthetic spread, backflow, or excessive chemosis due to altered anatomy from the band or scar tissue. Adjustments were made if necessary, and top-up injections were applied when required.

Patients were continuously monitored using pulse oximetry, 3-lead ECG, and non-invasive blood pressure measurement. All patients received an intravenous line with a Ringer’s acetate infusion. Analgosedation was administered to ensure patient comfort, with medications titrated as needed. These included propofol (10–20 mg boluses), dexmedetomidine (8–12 mcg boluses), fentanyl (25–50 mcg boluses), and ketamine (0.1–0.15 mg/kg bolus based on lean body weight), though their use was not standardised. In some cases, the STB was combined with GA to reduce postoperative opioid use and provide sustained analgesia. For general anesthesia, a premedication of 0.3 μg/kg bodyweight of dexmedetomidine iv was followed by propofol-alfentanil-ketamine induction, propofol TIVA maintenance (mg/kg/min) was used, and a laryngeal mask was placed to ensure airway patency in the majority of patients.

#### 2.3.2. Assessment of Technical Difficulty

Following block placement, the clinician rated the technical difficulty encountered due to scar tissue on a standardised scale from 1 to 3 (1 = easy, 2 = moderate, 3 = difficult). Chemosis (swelling of the conjunctiva) post-STB was also rated on a scale from 1 to 3 (1 = no chemosis, 2 = intermediate, 3 = heavy chemosis), providing insights into potential complications from the procedure. Block success was evaluated at the start and end of the procedure, specifically noting whether adequate anesthesia was achieved without the need for opioid supplementation in patients under GA.

Patient satisfaction rate was measured the first time immediately after surgery capturing the patient’s experience of anesthesia adequacy and comfort during the procedure, and again 6 to 24 h postoperatively on a scale from 1 to 5 (1 = very dissatisfied to 5 = completely satisfied). This satisfaction gauge was presumed to be reflective of postoperative analgesia.

### 2.4. Statistics

Descriptive statistics were used to summarize patient demographics, procedural details, and outcomes. Continuous variables, such as patient age and time since encircling band surgery, were presented as means with standard deviations (SDs) for normally distributed data or medians with interquartile ranges [IQRs] for non-normally distributed data. Categorical variables, including sex, STB success rates, and complication rates, were summarized as frequencies and percentages.

The primary outcome, the success rate of sub-Tenon’s block, was calculated as the proportion of patients for whom the block provided sufficient anesthesia without requiring conversion to another form of anesthesia or additional opioid use in cases combined with GA.

Comparisons between groups based on the technical difficulty of block placement (easy, moderate, or difficult) were performed using appropriate statistical tests. For categorical variables, we used the chi-squared test or Fisher’s exact test when expected cell counts were small. For continuous variables, the Kruskal–Wallis test was employed to compare non-normally distributed data across difficulty groups. Post hoc pairwise comparisons were conducted when significant differences were identified.

To assess the association between technical difficulty and block success, logistic regression analysis was performed, adjusting for potential confounders such as patient age and sex. Statistical significance was set at a *p*-value of <0.05. All analyses were conducted using SPSS, V.27 (IBM^®^, Armonk, NY, USA).

## 3. Results

We included 46 patients (37 males and 9 females) who underwent a total of 48 vitreoretinal surgeries (see Table 1). The average patient age was 61 years (range: 20–90 years). Surgical procedures included pars plana vitrectomy (PPV), oil removal, and complex phacoemulsification. The mean duration from encircling band surgery was 5.6 months, ranging from 6 days to 13 months, with one outlier at over 240 months.

### 3.1. Primary Outcome

The STB was successful in most cases (*n* = 43, 93.5%), meeting the primary objective of the study. Two cases required conversion to peribulbar block (PBB) due to extensive scarring, and one patient necessitated general anesthesia (GA) because of alcohol withdrawal-related agitation. Among the successful cases, the block provided sufficient anesthesia to complete the procedure without additional opioid administration.

### 3.2. Secondary Outcomes

**Figure 2:**
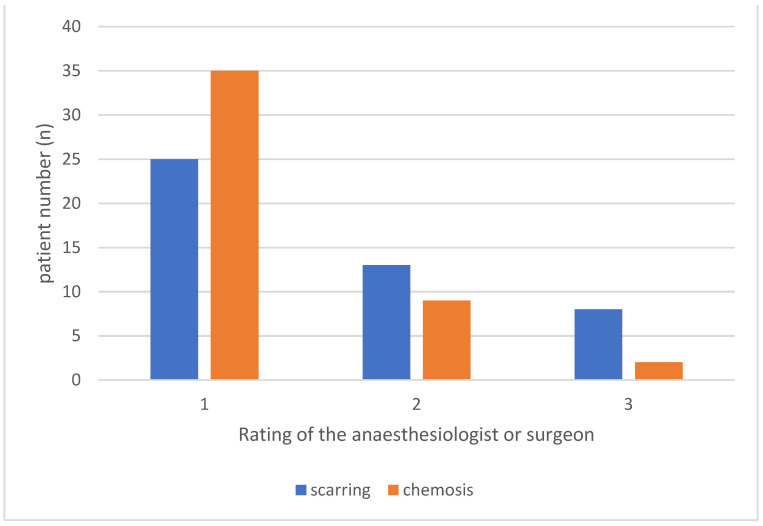
Scarring scaled from 1 to 3 (1 = easy, 2 = moderately difficult, 3 = difficult placement), chemosis after STB-administration also rated from 1 to 3 (1 = no chemosis, 2 = intermediate; 3 = heavy chemosis.

The safety objective was addressed through the analysis of complications. Chemosis was observed in 24% of patients, with severe cases reported in only 4%. No incidents of globe perforation or other major complications were recorded (consult Figure 2).

The surgeon performed the STB in 29 cases (63%), while an experienced anesthesiologist (FL) conducted the block in 19 cases (37%). Among these, the anesthesiologist independently administered the block in 17 cases, with the VR surgeon performing a top-up in 2 cases toward the end of the surgery. Clinician-reported technical difficulty ratings supported the feasibility objective. STB placement was rated as “easy” in 55% of cases, “moderately difficult” in 28%, and “difficult” in 17%. Higher difficulty ratings were associated with extensive scarring or band-related anatomical alterations. Chemosis occurred following block placement in 24% of cases, with severe chemosis observed in only two patients (4%). No statistical differences in performance were seen when the block was performed by the anesthetist vs. the surgeon (*p* = 0.405).

We also performed univariate regression analysis of the factors that influence block success. The results emphasize the importance of block difficulty and the absence of severe chemosis in determining the success of STB. Factors such as patient age and sex did not independently influence success, while the need for additional analgesia was indicative of reduced block efficacy (Table 2).

A multivariate logistic regression model was not feasible due to the small sample size of 46 patients and the limited number of unsuccessful blocks (*n* = 3), which did not meet the requirement of at least 10 events per predictor variable. The skewed distribution of outcomes (93.5% success rate) further limited the model’s ability to assess multiple predictors reliably. Sparse data in certain categories, such as “difficult” blocks and severe chemosis, compounded the issue by resulting in unstable estimates. To avoid overfitting and unreliable conclusions, we chose univariate logistic regression only to evaluate individual predictors.

Most patients (67%) received STB with analgosedation for both the block administration and the surgery, while 32% underwent general anesthesia (GA) in addition to the STB. A titrated approach was used for sedation, including propofol (mean dose 58 mg), fentanyl (mean 68 mcg), and dexmedetomidine (mean 11 mcg), with ketamine used in six cases (mean 16 mg). Only one patient required unplanned GA due to agitation from alcohol withdrawal; this case had a scar tissue rating of 3/3.

**Figure 3:**
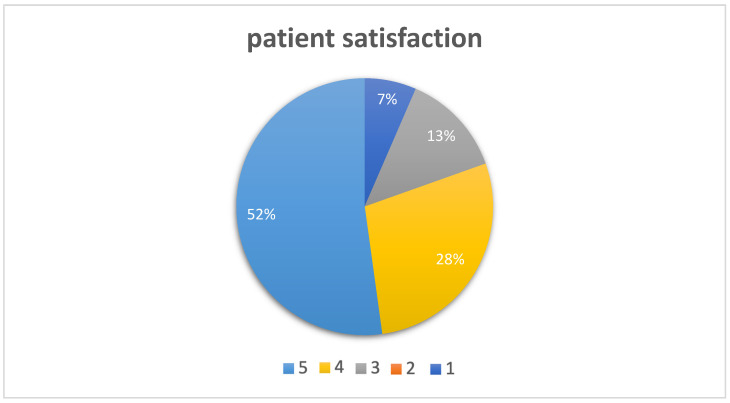
Patient satisfaction after surgery, 5 = completely satisfied, 4 = very satisfied, 3 = satisfied, 2 = dissatisfied, 1 = very dissatisfied.

Overall, patient satisfaction with the anesthesia technique was high (Figure 3); patient satisfaction, a secondary objective, was high, with a median satisfaction score of 5 (on a scale of 1–5) reported immediately postoperatively. However, patients with more challenging blocks demonstrated slightly lower satisfaction scores, reflecting the impact of technical difficulties on patient experience.

Most patients reported being very satisfied or satisfied, affirming STB as a feasible and effective approach for this patient cohort.

## 4. Discussion

This study provides preliminary and descriptive data on the use of sub-Tenon’s block (STB) in patients with prior encircling band surgery, highlighting its feasibility and safety. The STB effectively provided adequate anesthesia for vitreoretinal surgeries in most patients with prior encircling band surgery, with a low failure rate. Only two cases required additional intervention, and just one case needed unplanned conversion to general anesthesia. The administration of STB in patients with altered ocular anatomy due to scleral buckling was feasible, with 55% of cases rated as “easy” for block placement, 28% as “moderately difficult”, and only 17% as “difficult”. Severe chemosis was rare, occurring in just 4% of cases. The majority of patients expressed high satisfaction with the procedure, indicating that STB with or without sedation is a well-tolerated and preferred anesthesia option for this specific group, even with the anatomical complexities involved. These results suggest that STB is a viable alternative to GA, even in patients with altered ocular anatomy from prior surgery.

With regards to the primary outcome, we can state that a high success rate of STBs over indwelling encircling bands amounts to preliminary proof of feasibility. As for secondary outcomes, patient satisfaction was high with regards to intra- and postoperative pain. It was suggestive that patients with extensive scarring obtained insufficient analgesia. It can only be surmised that the two patients needing top-ups at the end of their surgery probably had patchy distributions of their blocks caused by encircling bands.

The use of STB in myopic eyes with previous scleral buckling poses unique challenges. Altered anatomy, particularly elongation of the globe, increases the risks associated with sharp-needle blocks, such as retrobulbar or peribulbar techniques. Dissection of scar tissue can lead to bulbar injury if strict visualization of ocular layers is not painstakingly pursued [9]. Increased bulbar length and the encircling band create technical difficulties, potentially raising the risk of complications like globe perforation. Compared to these sharp-needle methods, the blunt cannula used in STB allows for safer administration of local anesthetic, minimizing the risk of needle-related injuries (see Figure 1). STB can be thought of as the only intrafascial block providing sufficiently reliably regional anesthesia for surgery. The impediment of the encircling band to free intrafascial flow towards sensible nerves of the eye most likely favours patchy blocks, as they often occur in other intrafascial regional anesthesia [10].

In our cohort, STB administration was generally well-tolerated, with no incidences of apnea or bradycardia during liberal sedation. The only exception involved a patient in alcohol withdrawal who developed delirium, requiring a switch to GA. While the sedative requirements were slightly higher than usual, the surgery proceeded undisturbed in most cases. Importantly, this study emphasizes the need for careful patient selection and meticulous technique when applying STB in eyes with prior encircling surgery.

Despite the promising results, anatomical considerations merit further discussion. The encircling band can alter the distribution of local anesthetic, potentially creating “patchy” blocks in the sub-Tenon’s space divided by scar tissue or the band itself. This highlights the importance of having a surgeon and anesthesiologist prepared to address intraoperative pain with systemic medications, top-up local anesthesia, or alternative techniques like PBB.

Additionally, when STB is used in combination with GA for postoperative analgesia, clinicians must monitor hemodynamic changes carefully. The transient increase in intraocular pressure (IOP) caused by the local anesthetic depot in the sub-Tenon’s space could temporarily reduce retinal perfusion pressure, particularly in hypotensive patients [11]. Effective communication between anesthetists and surgeons is essential to identify and mitigate retinal perfusion deficits during surgery [12]. This hemodynamic concern may be more pronounced in previously encircled eyes due to their smaller sub-Tenon’s compartments, warranting further investigation.

The role of ultrasound (US) in regional ophthalmic anesthesia, particularly in STB, warrants further exploration. While US is invaluable in sharp-needle techniques, such as retrobulbar and peribulbar blocks, for avoiding complications like scleral or staphyloma puncture in altered ocular anatomy, its benefits in STB remain debated [13,14]. The encircling band’s contour is often visible under the conjunctiva and scar tissue, or it can be identified by tactile feedback using a conjunctival probe, negating the need for US guidance. Additionally, the band can be laid bare under a microscope in the surgical setting for further visualization. Incorporating US into STB poses unique challenges, including difficulty in visualizing the curved cannula, the thin tissue layer between the probe and cannula, and the risk of artifacts caused by metal and plastic components. These practical considerations, combined with the already high success rate of STB in this study, suggest that routine use of US may not be necessary. However, future research could evaluate whether US provides significant benefits in complex cases or for less experienced clinicians [5,15].

Overall, our findings suggest that STB is a feasible and safe alternative for patients with prior encircling band surgery. However, the technical challenges and physiological considerations underscore the importance of clinician expertise and real-time intraoperative management [16]. Further studies are needed to validate these findings and address the potential limitations posed by altered anatomy in this unique patient population.

The myopic eye poses distinct risks in the planning of locoregional anesthesia, especially sharp-needle blocks. Increased bulbar length is a significant limitation for retrobulbar blocks, as angulation of the needle with the orbital rim as a bevel point may increase the risk of bulbar perforation in the back of the eye.

Discussing our results in light of the preexisting literature is difficult given the prevailing scarcity of papers addressing this specific problem. Prior encircling is an independent risk factor for globe penetration, as found in a retrospective study evaluating this complication in patients undergoing retrobulbar and peribulbar blocks [7]. One case reported globe perforation during scissor-dissection of tissue while undertaking sub-Tenon’s in a previously encircled eye [9].

This study had limitations: As a retrospective cohort study, it is subject to biases related to data collection and lacks the control over variables that a prospective study would offer. This limits the ability to draw definitive causal inferences. With only 46 patients included, the sample size is relatively small, which may reduce the generalizability of the findings and limits the power to detect rare complications or differences across subgroups. This study was conducted at a single institution, which may limit the applicability of the results to other settings with different patient populations, clinician expertise, or procedural protocols. The STB was performed by both vitreoretinal surgeons and anesthesiologists, which introduces variability in technique and experience. This may influence the observed success rates and complication rates of the procedure. This study primarily focuses on intraoperative outcomes and immediate postoperative satisfaction without assessing long-term outcomes or potential late complications associated with STB in patients with previous encircling band surgery.

These limitations suggest that further research, ideally through a larger, multicenter prospective study, would be beneficial to validate these findings and better understand the risks and benefits of STB in this patient population.

## 5. Conclusions

In conclusion, this study provides valuable preliminary data supporting the feasibility and safety of sub-Tenon’s block (STB) as an effective anesthesia option for patients with prior scleral buckling surgery. Despite the anatomical challenges posed by scleral buckling, STB demonstrated a low failure rate, with the majority of patients experiencing successful block placement and high satisfaction with the procedure. The use of STB offers a viable alternative to general anesthesia (GA) with minimal complications.

However, the technical difficulties and potential for altered anesthetic distribution due to scar tissue or the encircling band highlight the importance of careful patient selection and expert execution. Additionally, while the STB technique was generally well-tolerated, clinicians must remain vigilant to the physiological challenges associated with altered ocular anatomy, particularly in managing intraoperative pain, hemodynamic changes, and retinal perfusion concerns.

Given this study’s retrospective nature, small sample size, and the single-center design, further investigation through larger, multicenter, and prospective studies is desirable to validate these findings.

## Figures and Tables

**Table 1:** Patient characteristics and detailed analysis of factors influencing or associated with the difficulty level of STB.

	STB Difficulty			*p*-Value	Total
	1 = Easy (*n* = 25)	2 = Moderate (*n* = 13)	3 = Difficult (*n* = 8)		46
Male Sex	19 (51.4)	11 (29.7)	7 (18.9)	0.701	37 (80.4)
Age	63.0 [51.5–71.5]	66.0 [60.5–69.0]	64.5 [50.25–71.25]	0.591	65.5 [56.25–70.25]
STB Success	25 (100)	13 (100)	5 (62.5)	**<0.001**	43 (93.5)
Chemosis				**0.011**	
None	21 (84.0)	11 (84.6)	3 (37.5)		35 (47.9)
Intermediate	4 (16.0)	2 (15.4)	3 (37.5)		9 (12.3)
Heavy	0 (0.0)	0 (0.0)	2 (25.0)		2 (2.7)
Patient Satisfaction	5 [4–5]	5 [4–5]	3.5 [1–5]	**0.028**	5 [4–5]
VAS during STB	1 [1–2]	1 [1–2]	4.5 [2.25–5.0]	**<0.001**	1 [1–2]
Administration of systemic analgesia	7 (28.0)	4 (30.8)	6 (75.0)	**0.049**	17 (23.3)
Switch to PBB	0 (0.0)	0 (0.0)	2 (25.0)	**0.007**	2 (4.3)
Switch to GA	0 (0.0)	0 (0.0)	1 (12.5)	0.088	1 (2.2)

Acronyms: **GA**: General Anesthesia; **PBB**: Peribulbar Block; **STB**: Sub-Tenon’s Block; **VAS**: Visual Analog Scale. Bold values significant for *p* < 0.005.

**Table 2:** Univariate logistic regression analysis of factors influencing sub-Tenon’s block success. OR = Odds Ratio; CI = Confidence Interval; NS = Not Significant. Reference category for block difficulty is “Easy”. Odds ratios represent the likelihood of block success compared to the reference group. A *p*-value of <0.05 indicates statistical significance. Predictors with “NS” were not significantly associated with block success.

Predictor	(OR)	95% (CI)	*p*-Value
Block Difficulty			
Easy (ref)	--	--	--
Moderate	0.85	0.55–1.30	**<0.001**
Difficult	0.15	0.05–0.45	**<0.001**
Chemosis Severity	0.40	0.18–0.89	**0.02**
Patient Age	NS	NS	>0.1
Sex	NS	NS	>0.1
Need for Additional Analgesia	0.30	0.10–0.85	**0.01**

Bold values significant for *p* < 0.005.

## Data Availability

The data presented in this study are available on request from the corresponding author. The data are not publicly available due to data protection and ethical regulations, as patients were not specifically consented for data-analysis by third parties.

## References

[1] Hamilton G.S. (2021). Aerosol-generating procedures in the COVID era. Respirology.

[2] Nouvellon E., Cuvillon P., Ripart J. (2010). Regional anesthesia and eye surgery. Anesthesiology.

[3] Kumar C.M., Seet E., Chua A.W.Y. (2023). Updates in ophthalmic anaesthesia in adults. BJA Educ..

[4] Chua M.J., Lersch F., Chua A.W.Y., Kumar C.M., Eke T. (2021). Sub-Tenon’s anaesthesia for modern eye surgery—Clinicians’ perspective, 30 years after re-introduction. Eye.

[5] Palte H.D. (2015). Ophthalmic regional blocks: Management, challenges, and solutions. Local Reg. Anesth..

[6] Kumar C.M., Dowd T.C. (2006). Complications of ophthalmic regional blocks: Their treatment and prevention. Ophthalmologica.

[7] Hay A., Flynn H.W., Hoffman J.I., Rivera A.H. (1991). Needle Penetration of the Globe during Retrobulbar and Peribulbar Injections. Ophthalmology.

[8] Chronopoulos A. (2015). Complications of Encircling Bands-Prevention and Management. J. Clin. Exp. Ophthalmol..

[9] Frieman B.J., Friedberg M.A. (2001). Globe perforation associated with subtenon’s anesthesia. Am. J. Ophthalmol..

[10] Juhl C.S., Rothe C., Støving K., Aasvang E.K., Rosenstock C.V., Lange K.H.W., Lundstrøm L.H. (2020). Intraindividual variation of the transversus abdominis plane block: An exploratory study in healthy volunteers. Reg. Anesth. Pain Med..

[11] Kelly D.J., Farrell S.M. (2018). Physiology and Role of Intraocular Pressure in Contemporary Anesthesia. Anesth. Analg..

[12] Sohn H.J., Moon H.S., Nam D.H., Paik H.J. (2008). Effect of volume used in sub-Tenon’s anesthesia on efficacy and intraocular pressure in vitreoretinal surgery. Ophthalmologica.

[13] Luyet C., Eichenberger U., Moriggl B., Remonda L., Greif R. (2008). Real-time visualization of ultrasound-guided retrobulbar blockade: An imaging study. Br. J. Anaesth..

[14] Sadler A., McLeod G., McHardy P.G., Wilkinson T. (2020). Ultrasound detection of iatrogenic injury during peribulbar eye block: A cadaveric study. Reg. Anesth. Pain Med..

[15] Lersch F., Schnidrig D., Boemke S., Djonov V., Jaggi D., Heussen F.M. (2024). Thiel cadaver eye as a training model for sub-Tenon’s blocks: A feasibility study. BMC Ophthalmol..

[16] Mavrakanas N.A., Stathopoulos C., Schutz J.S. (2011). Are ocular injection anesthetic blocks obsolete? Indications and guidelines. Curr. Opin. Ophthalmol..

